# Disentangling the local-scale drivers of taxonomic, phylogenetic and functional diversity in woody plant assemblages along elevational gradients in South Korea

**DOI:** 10.1371/journal.pone.0185763

**Published:** 2017-10-02

**Authors:** Jung-Hwa Chun, Chang-Bae Lee

**Affiliations:** 1 Division of Forest Ecology, National Institute of Forest Science, Dongdaemungu, Seoul, Republic of Korea; 2 Global Resources Division, Korea Forestry Promotion Institute, Gangseogu, Seoul, Republic of Korea; USDA Forest Service Rocky Mountain Research Station, UNITED STATES

## Abstract

Recently, new alternative matrices of biodiversity such as phylogenetic and functional diversity as a complement to species diversity have provided new insights into the mechanisms of community assembly. In this study, we analyzed the phylogenetic signals of five functional traits and the relative contribution of environmental variables and distance matrices to the alpha and beta components of taxonomic, phylogenetic and functional diversity in woody plant assemblages along four local elevational transects on two different mountains. We observed low but significant phylogenetic signals of functional traits, which suggest that phylogenetic dispersion can provide a rough approximation of functional dispersion but not perfect correlations between phylogenetic and functional diversity. Taxonomic alpha diversity showed a monotonic decline with elevation, and climatic variables were the main drivers of this pattern along all studied transects. Furthermore, although the phylogenetic and functional alpha dispersions showed different elevational patterns including increase, decrease and no relationship, the underlying processes driving the patterns of both types of alpha dispersion could be explained by the gradients of climatic and habitat variables as well as biotic interactions such as competition. These results suggest that both alpha dispersion patterns may be significantly controlled by niche-based deterministic processes such as biotic interactions and environmental filtering in our study areas. Moreover, the beta diversity with geographical distances showed distance-decay relationships for all transects. Although the relative importance of the environmental and geographical distances for beta diversity varied across the three facets of diversity and the transects, we generally found that environmental distances were more important for the beta components of the three facets of diversity. However, we cannot discriminate the effects of both distances on the three facets of diversity. Therefore, our study suggests that niche-based deterministic processes, potentially combined with neutral processes such as dispersal limitation and demographic stochasticity, may influence patterns of woody plant assemblage turnover in our study areas.

## Introduction

Quantifying the drivers shaping the spatial distribution and composition of biodiversity in ecosystems is a critical issue in ecology and biogeography. Many studies have considered the roles of environmental drivers in structuring diversity patterns in tropical, subtropical and temperate forests [1–4]. For example, the current climate as an imprint of historical and evolutionary factors is often correlated with species diversity patterns in studies spanning broader and larger geographic scales [5]. Moreover, species diversity and composition also vary along disturbance or habitat heterogeneity gradients at fine scales [6, 7].

However, such studies have mainly highlighted components of taxonomic diversity such as species richness, which treat all species as evolutionarily independent and ecologically equivalent and therefore may not convey enough information regarding the mechanisms underlying evolutionary histories or functional traits [8, 9]. For this reason, new alternative biodiversity matrices such as phylogenetic and functional diversity have been proposed during the past decade to improve our understanding of the drivers underlying biodiversity patterns, and ecologists have increasingly used these two different facets of biodiversity to provide new insights into studies on community ecology [10, 11]. Of these two new facets of biodiversity, phylogenetic diversity reflects the accumulated evolutionary and biogeographic history of a community [8], whereas functional diversity provides information regarding ongoing ecological processes inferred from morphological, physiological and ecological traits [11].

Although many hypotheses have been proposed to elucidate the contemporary distribution and composition of biodiversity [12], they fall within two main theories: the so-called niche-based deterministic processes versus the spatial-based neutral processes [8, 13, 14]. The niche-based deterministic hypothesis predicts that factors such as environmental filtering and biotic interactions (e.g., competition, facilitation and predation) play pivotal roles in regulating community assembly at local scales [13, 15], whereas spatial-based neutral theories emphasize the role of stochastic events such as dispersal limitation and local extinction in structuring species assemblages [13, 16]. Ecologists and biogeographers have widely used these competing theories to test the possible applications for explaining structuring community assembly in different regions. In addition, deterministic processes are generally expected to play a greater role in harsher environments [17].

In ecology, one of the most striking patterns of biodiversity is the decline in species diversity from the equator to the poles along latitude [18]. In addition, it is recognized that the latitudinal gradient in diversity is similar to the elevational gradient in diversity [19]. Although the two different gradients are substantially distinguishable in some respects [20], climatic-related factors, especially temperature-related variables, have similar patterns along both gradients and generally have considered that the primary drivers of the elevational diversity patterns are likely to be similar to those driving latitudinal diversity patterns [19]. Therefore, many researchers have recognized that the elevational gradient in mountain ecosystems offers the most powerful natural experimental system available to assess the ecological and evolutionary responses of organisms to environmental conditions [20–22]. Moreover, elevation has steeper environmental gradients than those along latitude for a given geographical distance [23]. Thus, studies of elevational gradients are more appropriate for testing diversity patterns driven by deterministic processes because steeper environmental gradients will increase the influence of deterministic processes in sorting species based on their respective niche requirements [24].

Although elevational diversity patterns have been a popular research subject in ecology and biogeography for two decades and there is ample evidence for the patterns and the associated underlying mechanisms affecting species [20, 21, 25] and phylogenetic diversity [26, 27], integrative analyses with three biodiversity facets including taxonomic, phylogenetic and functional diversity are very rare in previous studies on elevational diversity patterns. Moreover, most of the processes suggested to clarify the relationships between diversity and elevation have aimed to explain wide, large-scale patterns and do not completely explain the elevational diversity patterns uncovered at smaller scales such as local transects [28, 29]. However, diversity patterns can change depending on the spatial scale [21], and there is obviously a need to explore small-scale patterns [30]. The lack of such studies is partially due to the dependence on secondary distribution data from the literature as well as the abundance of proposed mechanisms in macroecology [28].

In this context, we investigated the taxonomic, phylogenetic and functional diversity patterns of woody plant assemblages along four local elevational transects on two mountains in South Korea. In addition, we also evaluated suitable variables that may explain these diversity patterns. Using data collected in a field survey, we focused on exploring 1) the patterns of taxonomic, phylogenetic and functional alpha and beta diversity along temperate elevational gradients; 2) whether these patterns differ between mountains with different peaks in elevation or transects on the same mountain; 3) which environmental variables (climate and habitat-related factors) can explain the alpha diversity patterns; and 4) which distance matrix (environmental and geographic distances) plays a more important role in shaping beta diversity.

## Materials and methods

### Study area and data collection

Our study was conducted along four transects on Mt. Seorak and Mt. Baekhwa (S1 Fig), which are typical rocky mountains of South Korea with randomly distributed rocky areas in each elevational band; the areas belong to a mountain ecoregion and a temperate, deciduous forest biome [31, 32]. Mt. Seorak is the third-highest mountain in South Korea and was designated the fifth Korean national park by Korean government in 1970 and the first biosphere reserve of Korea in 1982 by UNESCO. Mt. Seorak has an area of 398.2 km^2^ with the highest peak, Daechungbong, at 1708 m a.s.l., and the bedrock mainly contains dissected granite and gneiss [33]. The annual mean temperature and precipitation are 11°C and 1228 mm, respectively [32]. Mt. Baekhwa has an area of 15.9 km^2^ with the highest peak, Hansungbong, at 933 m a.s.l., and the bedrock is composed of granite and granite gneiss. The mean annual temperature and precipitation are 12.6°C and 1259 mm, respectively [31].

Mt. Seorak has four major types of vegetation along an elevational gradient, as follows: (1) temperate deciduous broad-leaf and pine forest (< 500 m) dominated by *Carpinus laxiflora*, *Lindera obtusiloba* and *Pinus densiflora*; (2) temperate broad-leaf and mixed forest (500–1100 m) dominated by *Quercus mongolica*, *Betula costata*, *Abies holophylla*, and *P*. *koraiensis*; (3) subalpine conifer forest (1100–1500 m) dominated by *Taxus cuspidata*, *A*. *nephrolepis* and *Juniperus chinensis* var. *sargentii*; and (4) alpine forest (> 1500 m) dominated by *P*. *pumila*, *Rhododendron mucronulatum* var. *ciliatum* and *B*. *ermanii* [32]. In addition, Mt. Baekhwa is divided into three vegetation types with elevation, namely, (1) warm temperate deciduous forest (< 350 m) dominated by *Q*. *serrate* and *Platycarya strobilacea*; (2) temperate deciduous forest (350–600 m) dominated by *Zelkova serrata* and *R*. *schlippenbachii*; and (3) temperate deciduous and pine forest (> 600 m) dominated by *P*. *densiflora* and *Acer pseudosieboldianum* [31].

We conducted field sampling from May to August of 2011 and 2012 along four transects on both mountains (Table 1). For the field survey, two 100-m-wide transects were established on each mountain up to the peaks along elevational gradients. Elevation was divided into elevational bands of 100 m intervals, and five plots were randomly established with a constant plot area of 400 m^2^ to control for sampling effort and area in each transect. Within each plot, plant species and cover-abundance scales were recorded following Braun-Blanquet [34]. We collected woody plant data from 130 plots on two transects on Mt. Seorak and 70 plots on two transects on Mt. Baekhwa. A total of 126 woody plant species were recorded including 44 families and 73 genera. The woody plant species and functional trait data at each elevational band are listed in the S1 Table.

**Table 1:** Characteristics of the four elevational transects (with abbreviations) used in this study.

Mountain	Study transect	Transect length (km)	Elevation extent (m)	Sampled domain (m)	No. of plots	No. of species
Mt. Seorak	Osaek (SOS)	5.3	457–1708	507–1676	60	72
	Siebisunnyutang (SSI)	22.4	235–1708	350–1687	70	91
Mt. Baekhwa	Banyasa (BBA)	3.5	269–933	300–925	35	66
	Bohyunsa (BBO)	4.1	255–933	301–925	35	58

### Phylogenetic tree

A phylogenetic tree containing all woody species was constructed using the online plant phylogeny software Phylomatic 3.0 [35], which uses the latest Angiosperm Phylogeny Group III (APG III) consensus tree (R20120829) as a backbone tree onto which species are added based on their taxonomy. Branch lengths were assigned to the tree using the BLADJ (branch length adjustment) function in Phylocom 4.2 [36] to constrain the internal nodes with available age estimates taken from Wikstrom et al. [37], and the other nodes were interpolated with unavailable age estimates [38]. The phylogenetic tree in this study is available in the S2 Fig.

### Functional traits and phylogenetic signal

We included five functional traits for all woody species: maximum height (*m*), leaf size (*cm*; length and width), flowering onset (*month*) and seed mass (*mg*). These functional traits are thought to represent important aspects of plant strategies and fundamental functional trade-offs [39, 40]. Maximum height is the dominant factor influencing access to light and represents a major axis of life history variation [41]. Leaf size has important consequences for leaf energy and water balance [39]. We used leaf length and width as a proxy of leaf area, which is the commonly used trait for leaf size, because we could not find data sources for leaf area of woody plant species for this study. Seed mass is an indicator of dispersal and regeneration strategy and represents the trade-off between the amount and size of seed [42]. Flowering onset is related to phenological and reproductive strategies and represents the trade-off between maximizing fruit set and reducing the risk of damage especially in temperate forests [40]. We obtained trait values for all woody species in the dataset from the literature and accessible online databases as described in the S2 Table. When more than one value was available for a given species, a mean value was calculated, and each species in the dataset was assigned the mean trait value. Therefore, our study does not explain the intra- and inter-specific trait variations. The values of all traits were log-transformed to improve normality and standardized before analysis. To eliminate trait redundancy, we performed principal components analysis (PCA) of the functional trait data. We used the four principal components, which explained 94.6% of the variation in the trait data, to construct an Euclidean trait distance matrix. An unweighted pair group method with arithmetic mean (UPGMA) with hierarchical clustering was then applied to this matrix to produce a trait dendrogram. The UPGMA dendrogram based on functional traits in this study is available in the S2 Fig.

One of the main aims in this study was to compare and contrast measurements of phylogenetic and functional diversity. Therefore, we quantified the phylogenetic signal of each functional trait using Blomberg’s *K* [43] and Pagel’s λ [44] to better understand the degree to which phylogenetic relatedness can estimate functional trait similarity of species. Blomberg’s *K* > 1 indicates stronger similarity among related species than expected by Brownian motion (BM), while *K* < 1 means less similarity among related species than expected under BM. Pagel’s λ = 0 implies no phylogenetic signal, and λ = 1 indicates that the distribution of a trait completely conforms to BM. To test the significance of the *K* and λ statistics, we randomly arrayed the trait data on the community phylogeny 1000 times to generate a null distribution.

### Calculations of alpha and beta diversity

The number of species in a plot was used for taxonomic alpha diversity, and the species turnover between plots was calculated using the Bray-Curtis similarity index for taxonomic beta diversity. The abundance-weighted net relatedness index (NRI) [8] was used to quantify phylogenetic and functional alpha and beta diversity. The formula is as follows:

$$NRI=\frac{-1\times \left(MPD_{sample}\;–\text{mean }MPD_{null}\right)}{sd\;MPD_{null}}$$

where MPD_sample_ is the observed mean phylogenetic distance (MPD) in a plot or between plots, mean MPD_null_ is the mean MPD of the null models and sd MPD_null_ is the standard deviation (SD) of the MPD of the null models. The MPD mainly reflects the deep phylogenetic structure of a phylogeny. That is, the MPD is usually thought to be more sensitive to the tree-wide patterns of phylogenetic clustering or overdispersion [8, 45] than to the structure near the tips. As the deeper parts of the phylogeny used are well supported and are derived from the APG III classification, there should be no significant bias in the NRI measurements caused by limited tip resolution in the phylogeny used. Functional diversity was calculated in the same way as the NRI [45] but using the functional dendrogram described in the “*Functional traits and phylogenetic signal*” section. We addressed that the observed phylogenetic or functional alpha and beta diversity deviated from the random expectation by comparing the observed values with 1000 null values generated by a null model. The null model randomly shuffled the names of species across the tips of the phylogenetic tree or functional trait dendrogram 1000 times. This approach randomized the phylogenetic or functional trait relatedness of species to one another while maintaining the observed community data matrix. Therefore, this null model fixes the observed levels of species alpha diversity, beta diversity, occupancy rates, abundance and spatial distributions in each randomization [9, 46]. A positive NRI value indicates that the observed MPD value is lower than that expected by chance and thus phylogenetic clustering occurs. In contrast, a negative NRI values indicates that the observed MPD is greater than that expected by chance and thus phylogenetic overdispersion occurs.

### Environmental variables

We included the mean annual temperature, the mean temperature in January (the coldest month), the mean temperature in August (the hottest month), the mean temperature of the growing season (May–August), the difference in temperature between January and August, the mean annual precipitation and the mean precipitation of growing season as climatic variables in each plot using national digital climate maps produced by the National Center of AgroMeteorology, Korea Meteorological Administration [47, 48]. The spatial resolutions of the raster data were 30 m and 270 m for temperature- and precipitation-related variables, respectively. In addition, temperature-related data were from 1971 to 2008, and precipitation-related data were from 1981 to 2009. PCA of all climatic variables was conducted to reduce co-variation and possible redundancy in the data. Two precipitation-related variables were log-transformed to achieve normality before the PCA. We used the first two PCA axes, which retained 96.9% of the total variance from the original variables, as new climatic variables (S2 Table). The PCA-derived climatic variables were named PC1_clim_ and PC2_clim_.

In addition to the climatic variables, we also measured habitat-related variables such as slope and rocky area ratio (RAR) in each plot with field measurements. Although edaphic variables including soil moisture and nutrient content are frequently used as substitutes for habitat variables in other studies [26, 29], we used slope and especially RAR because there are many rocky areas on both mountains, and a large proportions of these areas is located along the elevational gradients on the four transects, making it difficult to collect soil samples. Many plants including woody and herbaceous species in both mountains do not grow in such rocky locations [31, 32]. Therefore, we assumed that our variables would be more important for the occurrence and growth of woody plant species than other habitat-related variables. We measured the slopes from the center and the four corners of each plot using a clinometer and averaged the slopes by plot. To calculate the RAR, we placed four 50-m lines that penetrated the center of the plot, divided the lines into 1-m segments and recorded the proportion of various substrates (such as soil and rock) intercepting each segment. The RAR was calculated as the proportion of rocky substrate among 200 1-m segments in each plot. The slope was log-transformed, and the RAR was arcsine square root-transformed before analysis. The relationships between environmental variables and elevation along the four transects are shown in the S3 Fig.

### Statistical analysis

To test the effects of individual variables such as PC1_clim_, PC2_clim_, slope and RAR on elevational patterns of the alpha diversity components, we performed a simple ordinary least squares (OLS) regression analysis. Moreover, to investigate the relationships between the beta diversity components and geographical or each environmental distance, the geographical distance was calculated under an equal-distance projection (equidistant cylindrical; unit: m) using Euclidean distance measurements. In addition, we also calculated climatic distance with PC1_clim_ and PC2_clim_ and habitat distance with the transformed slope and RAR using Euclidean distance measurements. Then, a simple Mantel test was applied to evaluate the significance of the correlations between the distance matrices and the beta diversity components. We also used multiple OLS models and a model-averaging approach to assess the relative roles of the environmental variables or distance matrices in shaping patterns of alpha or beta diversity components. Model averaging was based on an information-theoretic approach that examined several competing hypotheses simultaneously to identify the best set of models via information criteria such as the Akaike’s information criterion (AIC) [49]. In this procedure, the relative importance of each environmental variable or distance matrix was quantified by summing the Akaike weights across all possible models containing the environmental variables or distance matrices.

Studies focusing on the mechanisms driving diversity patterns generally apply multiple regressions and similar statistical analysis [50]. However, more complex strategies are applied for ecological data analyses because it is important to explain the lack of independence between pairs of observations across the geographical space [51]. Therefore, in this study, we also used variation partitioning [52] using partial regressions for the alpha diversity components with environmental variables and multiple regressions on distance matrices (MRM) for the beta diversity components with distance matrices. Before the MRM, we calculated the environmental distance by integrating climatic and habitat distances based on the Euclidean distance measurements because one of the aims was to evaluate the relative importance of geographical and environmental distances on beta diversity components in this study.

## Results

### Phylogenetic signal

Although Blomberg’s K and Pagel’s λ values for each functional trait were less than 1, all traits exhibited significant phylogenetic signals (Table 2). These results suggest that using phylogenetic distance as a surrogate for differences in functional traits is appropriate for woody species along the four elevational transects in this study.

**Table 2:** Results from the phylogenetic signal test for each functional trait from the study transects using Blomberg's *K* and Pagel’s λ statistics.

Functional trait	Blomberg’s *K*	Pagel’s λ
Tree height (m)	0.414***	0.834***
Leaf length (cm)	0.314***	0.684***
Leaf width (cm)	0.620***	0.799***
Flowering onset (month)	0.241*	0.528*
Seed weight (mg)	0.623***	0.998***

Significance levels are * *P* < 0.05, ** *P* < 0.01, *** *P* < 0.001. The abbreviations for study transects are as described in Table 1.

### Alpha and beta diversity along elevational gradients

**Fig 1:**
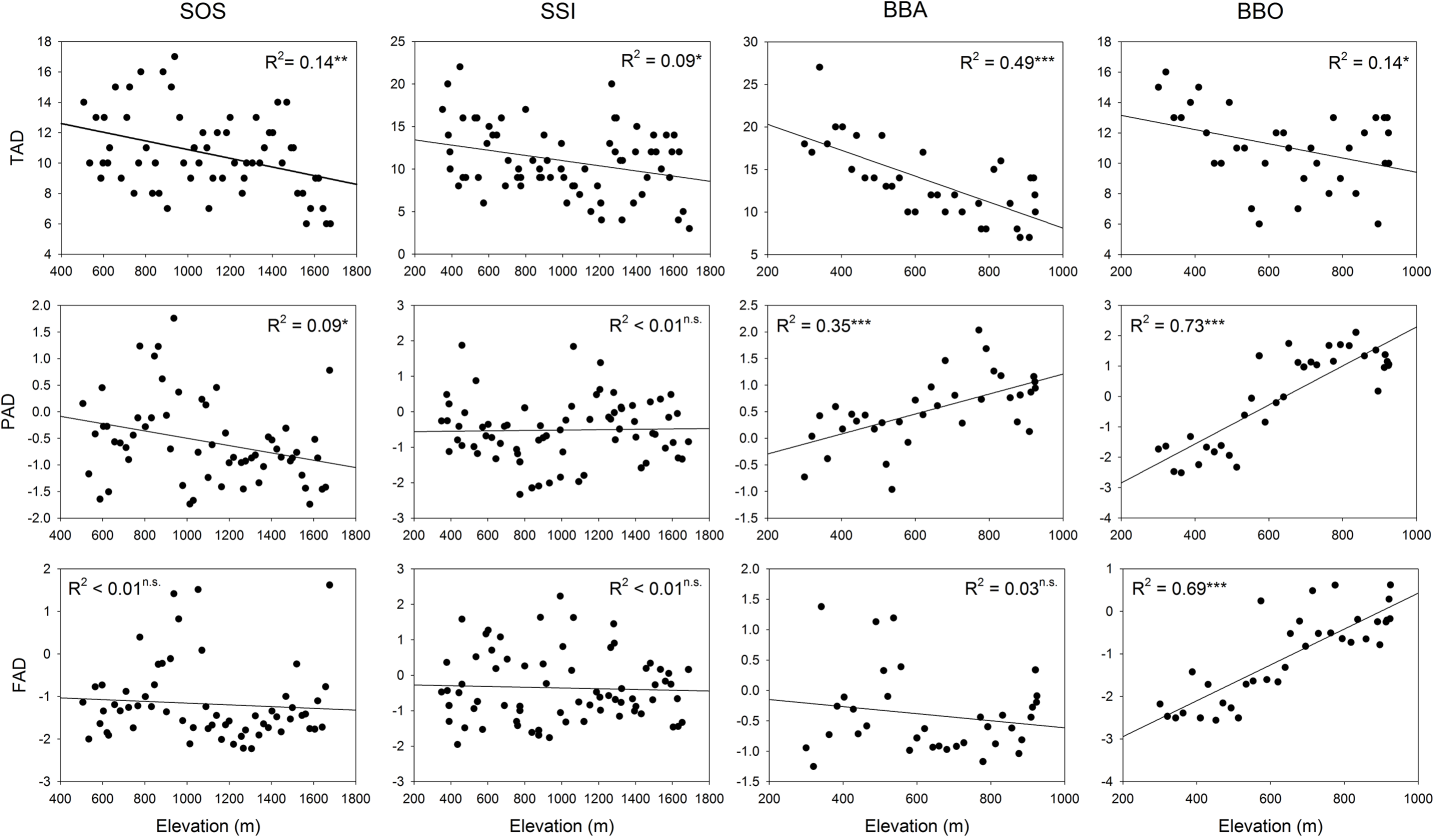
Relatio*n*ships between elevation and three alpha diversity components along the four transects.

**Fig 2:**
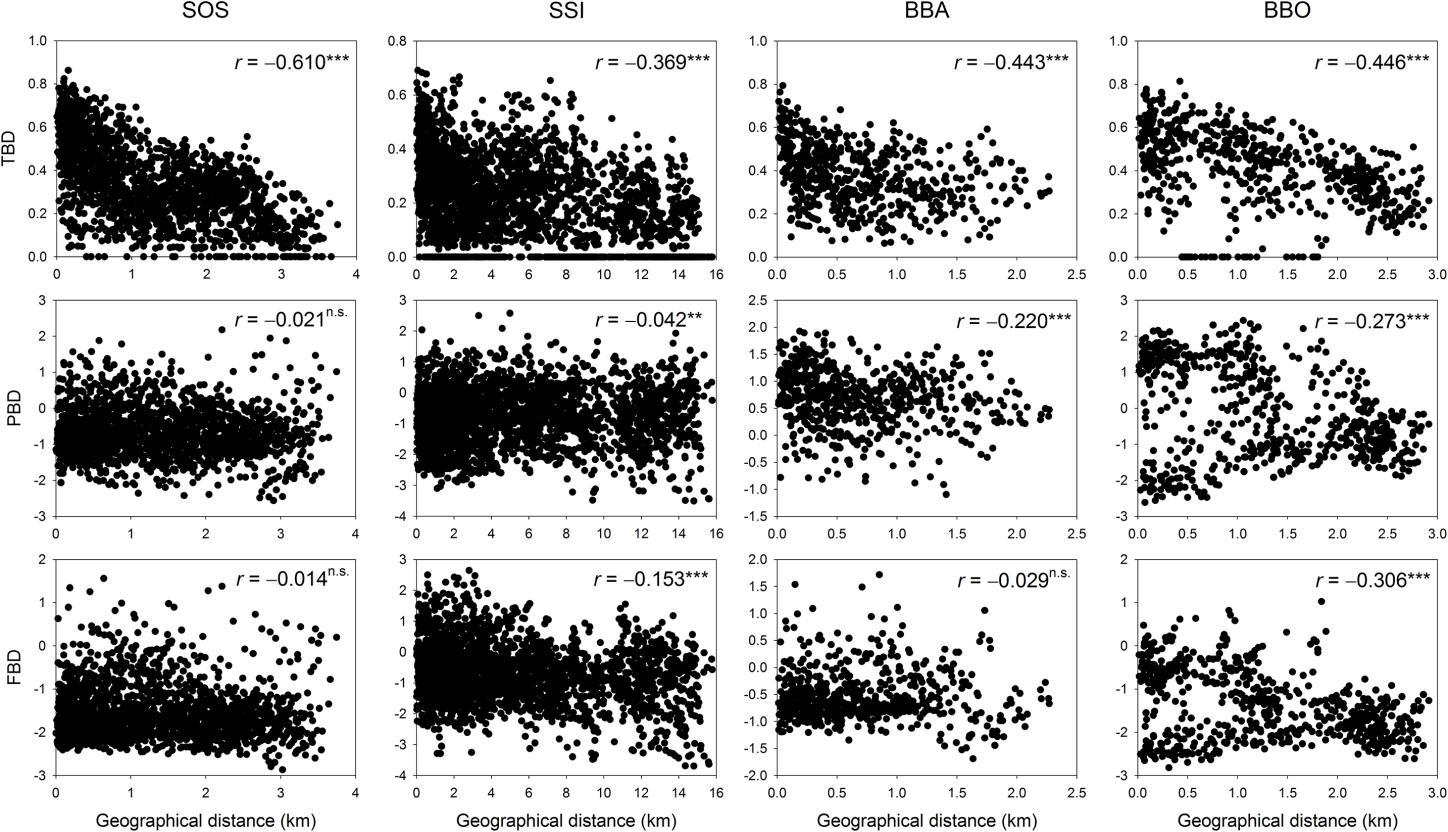
Relationships between geographical distance and three beta diversity components along the four transects.

Taxonomic alpha diversity based on the species richness of woody plants was negatively correlated with elevation for all study transects. However, the patterns of phylogenetic and functional alpha diversity were different among transects and included increase, decrease and no relationship (Fig 1). Overall, except for three case that showed no relationships (i.e., phylogenetic and functional beta diversity along the SOS transect and functional beta diversity along the BBA transect) the patterns of taxonomic, phylogenetic and functional beta diversity were negatively correlated with geographical distance along the study transects (Fig 2).

### Determinants of alpha and beta diversity

The OLS regression results indicated that overall, taxonomic and functional alpha diversity were primarily related to the climatic and habitat variables, respectively, whereas the relative importance of the environmental variables for phylogenetic alpha diversity was different among the study transects (Table 3). Simple Mantel tests revealed that the relative importance of geographical and environmental distances for taxonomic and phylogenetic beta diversity differed among the study transects (Table 4). In addition, the functional beta diversity was mainly controlled by environmental distance, such as climatic and habitat distances.

**Table 3:** Coefficient of determination (R^2^) and significance level from simple ordinary least squares regression models for environmental variables and three alpha diversity components along four elevational transects.

Transect	Diversity index	PC1_clim_	PC2_clim_	Slope	RAR
SOS	TAD	(+) 0.14**	(–) 0.17***	(+) 0.03	(–) 0.08*
	PAD	(+) 0.11**	(–) 0.10*	(–) 0.03	(–) 0.14**
	FAD	(+) 0.01	(–) < 0.01	(–) 0.11**	(–) 0.29***
SSI	TAD	(+) 0.17***	(+) 0.09*	(–) < 0.01	(–) 0.12**
	PAD	(+) < 0.01	(–) 0.01	(–) 0.12**	(–) 0.14***
	FAD	(+) < 0.01	(+) < 0.01	(–) 0.08*	(–) 0.29***
BBA	TAD	(+) 0.08	(+) 0.50***	(–) 0.10	(–) 0.10
	PAD	(–) 0.13*	(–) 0.31***	(+) 0.01	(–) < 0.01
	FAD	(–) 0.02	(+) 0.04	(–) 0.14*	(–) 0.21**
BBO	TAD	(+) 0.14*	(+) 0.11	(–) 0.03	(+) 0.02
	PAD	(–) 0.75***	(–) 0.69***	(+) 0.01	(–) 0.27***
	FAD	(–) 0.71***	(–) 0.62***	(+) < 0.01	(–) 0.38***

Significance levels are * *P* < 0.05, ** *P* < 0.01, *** *P* < 0.001. The symbols in parenthesis indicate the relationship between the diversity index and environmental variable. Abbreviations: TAD—taxonomic alpha diversity; PAD—phylogenetic alpha diversity; FAD—functional alpha diversity; RAR—rocky area ratio. The abbreviations for the study transects are described in Table 1.

**Table 4:** Results of the simple Mantel tests investigating the effects of geographical and environmental distances on three beta diversity components along four elevational transects.

Transect	Diversity index	*Dist*_*geo*_	*Dist*_*clim*_	*Dist*_*habit*_
SOS	TBD	–0.610***	–0.614***	–0.218***
	PBD	–0.021	–0.072***	–0.143***
	FBD	–0.014	–0.074***	–0.187***
SSI	TBD	–0.369***	–0.392***	–0.103***
	PBD	–0.042**	0.044**	–0.045**
	FBD	–0.152***	–0.153***	–0.115***
BBA	TBD	–0.443***	–0.318***	–0.149**
	PBD	–0.220***	–0.137***	–0.072*
	FBD	–0.029	–0.047	–0.057*
BBO	TBD	–0.446***	–0.439***	–0.177***
	PBD	–0.273***	–0.351***	–0.119***
	FBD	–0.306***	–0.369***	–0.107***

Significance levels are * *P* < 0.05, ** *P* < 0.01, *** *P* < 0.001. Abbreviations: TBD—taxonomic beta diversity; PBD—phylogenetic beta diversity; FBD—functional beta diversity; *Dist*_*geo*_–geographical distance; *Dist*_*clim*_–climatic distance; *Dist*_*habit*_–habitat distance. The abbreviations for study transects are described in Table 1.

**Fig 3:**
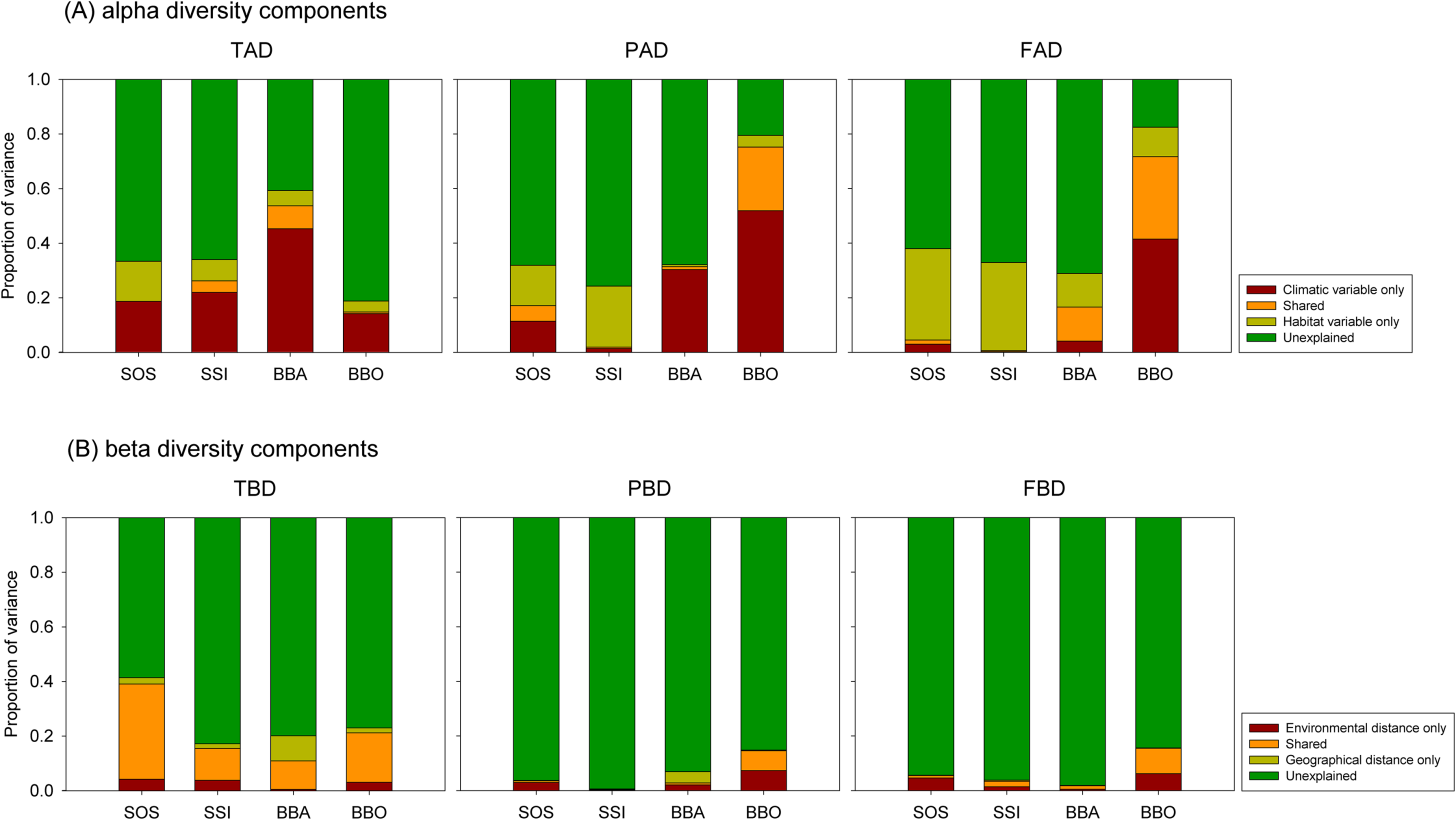
Variation partitioning for three alpha and beta diversity components by environmental variables and distance matrices, respectively, along the four transects.

The results from the multi-model inference with importance value were similar to those of simple OLS and simple Mantel tests. Climatic variables were the most important for taxonomic alpha diversity along the four transects, and habitat variables were the main factors governing the patterns of functional alpha diversity (Table 5). The crucial variables for phylogenetic alpha diversity were different among the four transects. These results were also similar to results from the variation partitioning analysis (Fig 3). The environmental distances were relatively important for the beta diversity components with two exceptions: taxonomic and phylogenetic beta diversity in BBA (Table 5 and Fig 3b). In addition, the relative importance between climatic and habitat distances differed among transects (Table 5 and S3 Table).

**Table 5:** Importance value of each variable based on multi-model inference in determining the alpha and beta diversity of woody plant assemblages along four elevational gradients. Importance values are the posterior probabilities over the set of hypotheses and represent the sum of the Akaike weights for each model containing a specific predictor in the model set.

**(A) alpha diversity**												
	**TAD**				**PAD**				**FAD**			
**Variable**	**SOS**	**SSI**	**BBA**	**BBO**	**SOS**	**SSI**	**BBA**	**BBO**	**SOS**	**SSI**	**BBA**	**BBO**
PC1_clim_	**0.757**	**0.999**	0.309	**0.645**	0.483	0.289	0.231	**0.991**	0.385	0.276	0.302	**0.998**
PC2_clim_	**0.955**	**0.978**	**1**	0.443	**0.706**	0.333	**0.975**	0.247	**0.474**	0.297	0.41	**0.504**
Slope	0.270	0.256	0.363	0.367	**0.588**	**0.897**	0.23	0.245	**0.795**	**0.684**	0.425	0.267
RAR	**0.980**	**0.924**	**0.713**	0.237	**0.984**	**0.971**	0.226	**0.82**	**1**	**1**	**0.846**	**0.998**
**(B) beta diversity**												
	**TBD**				**PBD**				**FBD**			
**Variable**	**SOS**	**SSI**	**BBA**	**BBO**	**SOS**	**SSI**	**BBA**	**BBO**	**SOS**	**SSI**	**BBA**	**BBO**
*Dist*_*geo*_	**1**	**1**	**1**	**0.997**	**0.994**	0.543	**1**	0.406	**1**	**0.989**	**0.921**	0.274
*Dist*_*clim*_	**1**	**1**	**0.573**	**0.979**	**1**	**0.613**	**0.595**	**1**	**1**	**0.946**	**0.926**	**1**
*Dist*_*habit*_	**1**	**0.995**	0.401	**0.997**	**1**	**0.859**	**0.988**	**0.999**	**1**	**1**	**0.92**	**0.997**

The variables included in the best model from all 15 (for alpha diversity) or 7 (for beta diversity) possible models are highlighted in bold, and the best models are based on minimizing the corrected Akaike information criterion among all possible models. The abbreviations are as described in Tables 1–4.

## Discussion

In this study, we explored small-scale elevational patterns of the alpha and beta components of three facets of biodiversity and their drivers on different mountains and along different transects using primary data; this study was thus unlike many studies that explore broad and large-scale patterns using secondary data. In addition, we also investigated the phylogenetic signals of five functional traits to better understand the degree to which the phylogenetic tree can estimate functional trait similarity of species. Primary data at a small scale are central to understanding the within-domain diversity in biogeographic groups, whereas large-scale secondary data are critical for understanding the patterns across spatial scales [28, 32]. Therefore, the advantage of the present study is the exploration of elevational patterns and processes using empirical data collected at local scales along with integrative analyses of three aspects of biodiversity (i.e., taxonomic, phylogenetic, and functional diversity).

### Phylogenetic signal of functional traits

The overall low phylogenetic signals of functional traits (i.e., Blomberg’s *K* and Pagel’s λ values < 1) suggest that high evolutionary trait lability rather than conservatism drives species sorting along the four elevational transects [8], and this simultaneously indicates that convergent adaptations occur in this temperate mountainous environment [53, 54]. In this study, we quantified the phylogenetic signals of the functional traits of five woody species along the four elevational transects. All trait values had significant phylogenetic signals when using a randomization test to assess significance (Table 2). The general congruence of the functional and phylogenetic dispersion is supported by the significant phylogenetic signal of the trait data, but the congruence is not complete between the two dispersion patterns in our study transects. This result may be explained by noting that the *K* and λ values for phylogenetic signal were less than 1, which indicates that the functional traits are more labile than expected under Brownian motion of trait evolution. Similar results were reported in other studies and indicated that phylogenetic and functional trait dispersions are aligned despite having imperfect and weak (but significant) phylogenetic signals of trait data [45, 55, 56]. Thus, our results suggest that if there are significant but low values of phylogenetic signals of functional traits, then phylogenetic dispersion can roughly estimate functional dispersion; however, there were not ‘perfect matches’ between both facets of diversity. Moreover, if there are phylogenetic clustering patterns in community assemblies of woody plants along elevational gradients in our study areas, our results may suggest the possibility of a direct association between phylogenetic clustering and environmental filtering as one of the deterministic processes structuring community assembly. In other words, the idea that success of the phylogenetic clustering approach is mainly derived from stronger environmental filtering rather than competitive exclusion relies on the assumption that the functional traits involved in community assembly processes have detectable phylogenetic signals [13, 14]. Indeed, many ecologists and biogeographers have increasingly recognized that checking the phylogenetic signals of functional traits is a key stage when implementing phylogenetic community structure analysis [8, 13, 14].

However, our results also raise the question of why these ‘not perfect matches’ occur between phylogenetic relatedness and functional traits. First, in general, phylogenetic relatedness is used as an indirect estimate of ecological similarity [11]. Therefore, the phylogenetic relatedness will still only represent an indirect proxy of the overall trait similarity of species in a community, and thus may not be able to uncover similarities in individual functional traits. Second, although the estimation of the general similarity for species is useful and tractable in some circumstances, this estimation is likely to average out important and appealing information relevant to one or few traits of species. Thus, much information can be lost or rinsed out when using phylogenetic relatedness as a substitute for trait similarity [57]. Moreover, an alternative problem is that community assembly and species coexistence may be primarily influenced by a single resource axis, and potentially only one functional trait is important for understanding the processes underlying community assembly; however, phylogenetic relatedness likely cannot detect such processes [11]. However, these irremovable limitations occurring due to the use of phylogenetic relatedness as a proxy for indirect ecological similarity can still occur when using functional traits as a substitute for direct ecological similarity. Many studies relevant to the functional trait approach, including our study, use a few easily measurable indirect traits and fundamental aspects of ecological strategy and functional trade-offs (e.g., morphological and structural traits, nutrient content) related to physiological processes in plants [9, 46]; this approach is used because it is impossible to measure all traits thought to be important for physiological and defense mechanisms. Moreover, we do not know which traits are important for evolutionary processes in community assembly, and the functional trait approach also has inherent weakness such as intra- and inter-specific variation [9, 11, 46]. These limitations and shortcomings between both phylogenetic relatedness and functional trait approaches are likely to result in imperfect congruence between both phylogenetic and functional dispersions. Therefore, our results re-emphasize that studies on structure of community assembly and the underlying processes must use both approaches complementarily, which has also been emphasized in previous studies [46].

### Plant diversity and drivers along elevational gradients

For taxonomic alpha diversity based on species richness, our study showed a monotonic decline patterns with increasing elevation along all study transects. Generally, monotonic decline patterns are recognized as one of the dominant types along elevational gradients. Rahbek [21] estimated that approximately 50%, 25% and 25% of the documented elevational patterns were unimodal, monotonic decreasing and other patterns, respectively. Moreover, many other studies have documented that climate is an obvious factor controlling species distribution and taxonomic diversity based on species richness in many areas [58, 59]. Therefore, our study adds to the growing evidence that taxonomic alpha diversity exhibits a pattern of monotonic decline along local transects in mountain ecosystems with changes in elevation, and climatic variables are one of main drivers of these patterns.

In contrast to taxonomic alpha diversity, the phylogenetic and functional alpha diversity showed different patterns (i.e., increase, decrease or no relationship) with increasing elevation among the transects. Along both elevational transects on Mt. Baekhwa, the phylogenetic alpha diversity of woody assemblages showed phylogenetic clustering patterns, whereas phylogenetic overdispersion and randomness were observed along the SOS and SSI transects on Mt. Seorak, respectively. The observed increasing pattern of phylogenetic relatedness along both elevational transects on Mt. Baekhwa may be explained by the fact that community assembly at high elevations was more derived from strong environmental filtering processes than competitive exclusion processes [8, 38]; this was especially true for climate-related filtering processes whereby closely related species with adaptive characteristics for harsh environmental conditions such as low temperature and strong wind are filtered [26, 60, 61]. In addition, the decreased phylogenetic relatedness at low elevations can be interpreted as evidence that the effect of competitive exclusion from competition between related species with ecological similarity is stronger than the environmental filtering effect; thus, these clades are distantly related to other temperate lineages at low elevations [13, 14]. Indeed, many genera were observed only in plots at low elevations along the BBA (15 genera including *Akebia*, *Corylus*, *Morus*, *etc*.) and BBO (11 genera including *Alnus*, *Juniperus*, *Lonicera*, *etc*.) transects, whereas a smaller number of genera (3 and 7 genera along the BBA and BBO transects, respectively) were recorded only in plots at higher elevations (> 600 m) along the two transects (S1 Table). These differences in unique genera distributed on low and high elevations represent phylogenetic clustering patterns along the two transects on Mt. Baekhwa.

The observed pattern of decline in phylogenetic relatedness with increasing elevation along the SOS transect on Mt. Seorak can be interpreted several ways. First, if competitive exclusion primarily removes related species with ecological similarity that overlap too strongly in their niches at high elevation and if the degree to which different species with superior traits preferentially occupy the limited resources at high elevation is positively related to phylogenetic distance, then competition will drive phylogenetic overdispersion [60, 62]. An alternative plausible explanation is related to climatic variables such as the temperature differences between the hottest and coldest months along the SSO transect in Mt. Seorak. The temperature difference between the hottest and coldest months is generally recognized as temperature seasonality or variability. The values along the SSO transect increase, which contrasts similar patterns of the other climate variables (i.e. mean annual temperature, monthly mean temperatures of the coldest and hottest months and mean temperature in the growing season) along the other transects (S3 Fig). This wider temperature spectrum at higher elevations along the SSO transect might allow some phylogenetically distant species, that are already adapted to cold conditions and wide ranges of temperatures across their evolutionary histories, to occur more frequently in high-elevation habitats than closely related species. Indeed, several genera such as *Hydrangea*, *Lonicera*, *Rosa*, *Syringa*, and *Thuja* are common at high elevation plots (> 1100 m) along the SSO transect (S1 Table), and these genera represent the contribution of completely novel lineages to the woody assemblages at higher elevations on this transect [32, 33]; many other genera are widely distributed across the entire elevation ranges along the SSO transect (S1 Table). However, these two plausible explanations are not mutually exclusive and independent and may be simultaneously involved in the phylogenetic overdispersion at higher elevations along the SSO transect. Along the SSI transect on Mt. Seorak, although the observed random phylogenetic structure suggests that neutral processes structure local communities, we cannot interpret this result as coming from spatial-based neutral processes because the pattern was closely related to the gradients of habitat variables such as slope and RAR (Tables 3 and 5). Our results revealed that phylogenetic dispersion along the SSI transect changes significantly along the gradients of habitat-related factors. Other studies have also shown that the gradients in habitat factors are important driving forces of the phylogenetic structure of community assemblies [4]. Generally, many studies on the elevational patterns of phylogenetic dispersion have demonstrated phylogenetic clustering patterns at higher elevations with the effect of abiotic filtering such as by environmental filters [15, 27, 56, 60, 61], whereas few studies revealed phylogenetic overdispersion due to the effects of biotic interactions such as interspecific competition at higher elevations [2, 3]. Although the patterns of phylogenetic dispersion and the underlying processes along elevational gradients differed among study areas in our study and in other studies, phylogenetic clustering at higher elevations seems to be a predominant pattern rather than overdispersion. However, the underlying mechanism is mainly explained by niche-based deterministic theory including abiotic (e.g., environmental filtering by climate or habitat gradients) and biotic (e.g., competition) processes for both dispersion patterns [17]. Similarly, although functional alpha diversity only showed a clustering pattern along the BBO on Mt. Baekhwa and there were no relationships along the other transects, the processes shaping the patterns of functional alpha diversity were related to the gradients of climatic and habitat variables along the BBO transect and mainly to the gradients of habitat variables along the other transects. These results also support the hypothesis that niche-based deterministic processes are fundamental for functional alpha diversity in woody community assemblies along our study transects, although the main abiotic filters are different among the study transects. Other studies have also documented the importance of climate [5] and habitat factors [56, 63] in determining the functional structure of woody plant assemblages. Although our study emphasizes the importance of niche-based deterministic processes for determining the patterns of phylogenetic and functional alpha diversity, the implications of the processes for the two diversity indices are different. That is, the processes associated with phylogenetic diversity patterns varied among the study transects, whereas the processes structuring functional diversity were mainly associated with the gradients of habitat variables in the study transects. These results suggest that phylogenetic diversity, which reflects the accumulated evolutionary and biogeographic history of community assembly, is linked to various abiotic and biotic processes. These results also suggest that functional diversity, which is related to the ongoing ecological processes inferred from morphological, physiological and ecological traits, is associated with a set of species with functional traits that are optimally adaptable for a given environment or set of habitat conditions irrespective of the phylogenetic distance among lineages.

We also evaluated whether the beta components of the three diversity facets between pairs of forest plots were related to their environmental and spatial distances. The beta diversity patterns with geographical distances showed distance-decay relationships, which describe the decrease in compositional similarity between two communities with increasing geographical distance between them [64]. Although the relative importance of environmental and geographical distances varied across the three facets of diversity and study transects, we found that environmental distances were relatively important for the three beta diversity components. However, we cannot discriminate the distinct effects among both distances on the three facets of diversity (Table 5 and Fig 3b), suggesting that deterministic processes such as ecological and evolutionary differentiation among species and biotic and abiotic filtering, potentially combined with stochastic events such as dispersal limitation and demographic stochasticity, may influence patterns of assemblage turnover [65, 66].

In summary, our study found weak but significant phylogenetic signals of functional traits along our study transects, which suggested that phylogenetic dispersion can provide a rough approximation of functional dispersion but not perfect matches between phylogenetic and functional diversity. Taxonomic alpha diversity showed patterns of monotonic decline along all transects and that the main drivers were climatic variables, whereas the patterns of phylogenetic and functional alpha diversity and their main drivers were different among the study transects. However, our results suggest that phylogenetic and functional alpha diversity patterns are structured by niche-based deterministic processes including biotic (e.g., competitive exclusion) and abiotic (e.g., environmental filtering by habitat and climatic factors) interactions but not by stochastic processes. For the beta diversity of the three facets, although environmental distances were relatively more important than geographical distances, it is difficult to distinguish the effects of both distances. In addition, these results indicate that niche-based deterministic and stochastic processes may simultaneously control the patterns of beta diversity in our study areas.

## Supporting information

S1 Fig Location and topography of the study transects on two mountains in South Korea. (TIF)

S2 Fig Images of the phylogenetic and functional trees of woody plant species from the four transects. (PDF)

S3 Fig Relationships between elevation and environmental variables along the four transects. The abbreviations for environmental variables are as described in the S2 Table.
(TIF)

S1 Table Functional trait data for woody plant species used in this study and the frequencies of species found at each elevational band along the four study transects. The scientific names of the plant species followed the National Plant Species Database System (http://www.nature.go.kr) published by the Korea National Arboretum, Korea Forest Service. Functional traits of woody plant species were obtained from the literature and public online trait databases, and the four traits except for seed weight, leaf length and width were obtained from the Korea Biodiversity Information System. Abbreviations: LF—life form; MH—maximum height; LL—leaf length; LW—leaf width; FO—flowering onset; SW—seed weight; T—tree; S—shrub; V—vine.
(XLSX)

S2 Table The results of the principal components analysis with seven climatic variables. (DOCX)

S3 Table Variation partitioning of the beta diversity components explained by climatic and habitat distances along the four elevational transects. The explained variance was divided into three parts: (1) pure effect of climatic distance; (2) shared effect of climatic and habitat distances; and (3) pure effect of habitat distance. The abbreviations for transect and diversity index are as described in Tables 1 and 4.
(DOCX)
