# Supplementary figures and images for: Disentangling the local-scale drivers of taxonomic, phylogenetic and functional diversity in woody plant assemblages along elevational gradients in South Korea
Source: PLoS One. 2017 Oct 2;12(10):e0185763. doi: 10.1371/journal.pone.0185763 (PMC5624625; doi:10.1371/journal.pone.0185763)

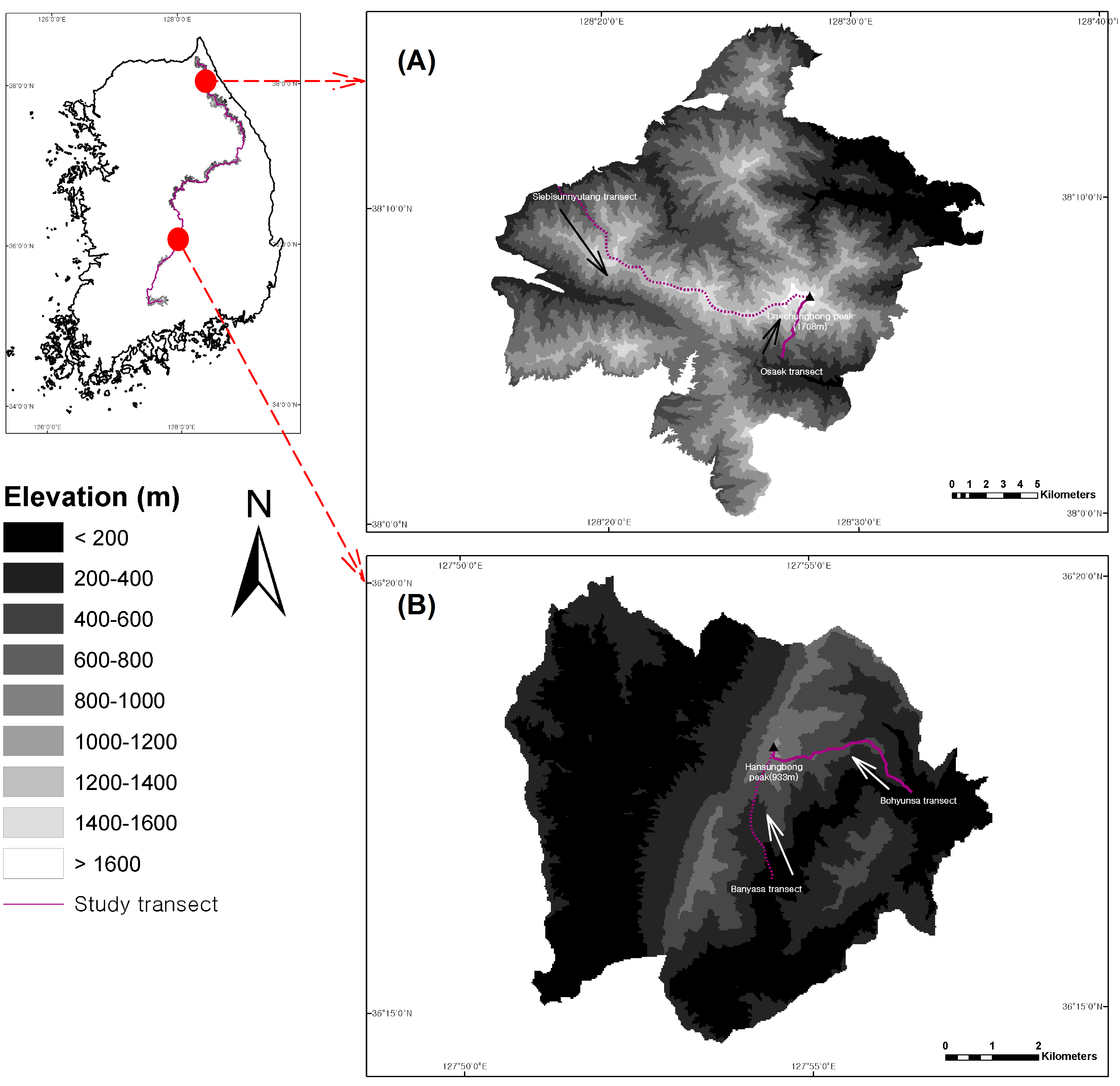

Supplement: S1 Fig — (TIF) [file pone.0185763.s001.TIF]

(A) Phylogenetic tree

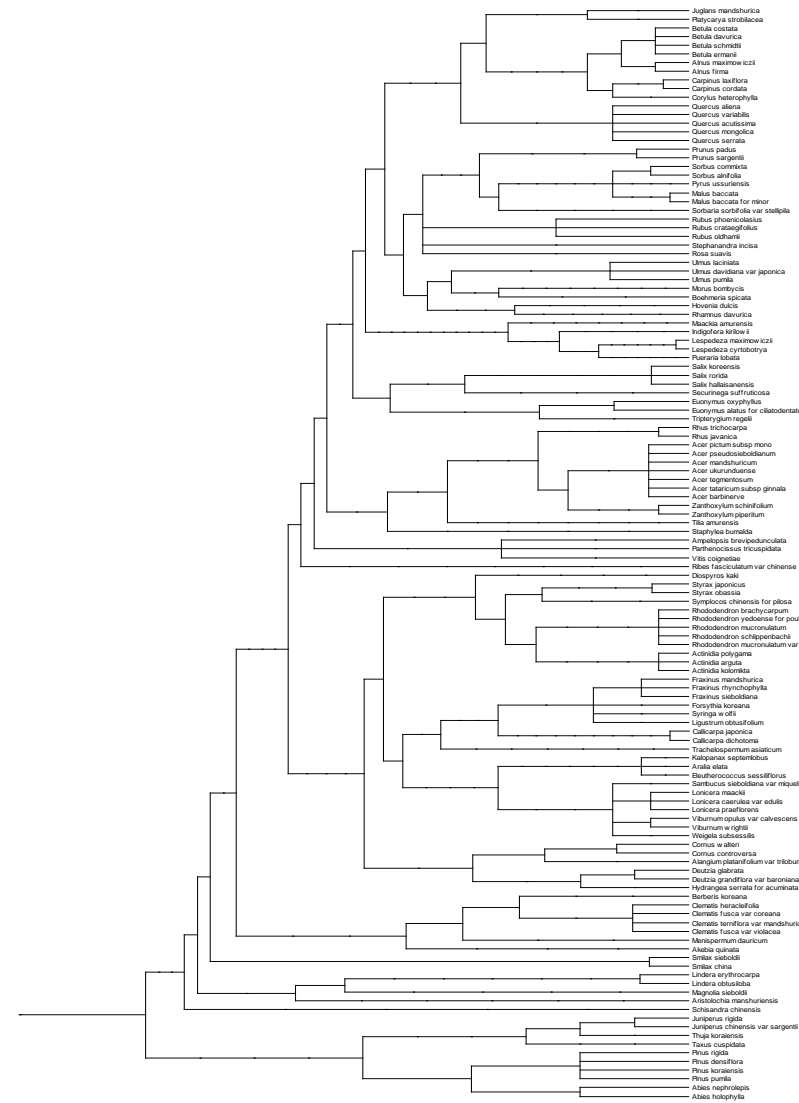

(B) Functional trait dendrogram

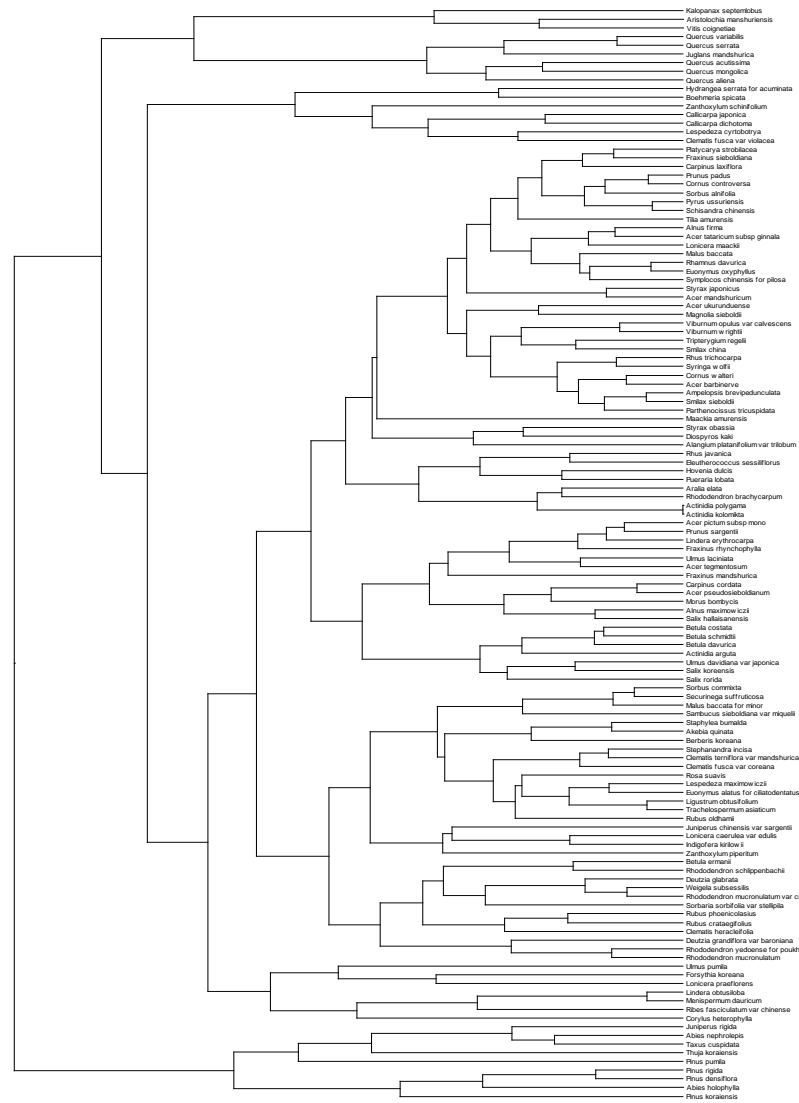

Supplement: S2 Fig — (PDF) [file pone.0185763.s002.pdf]

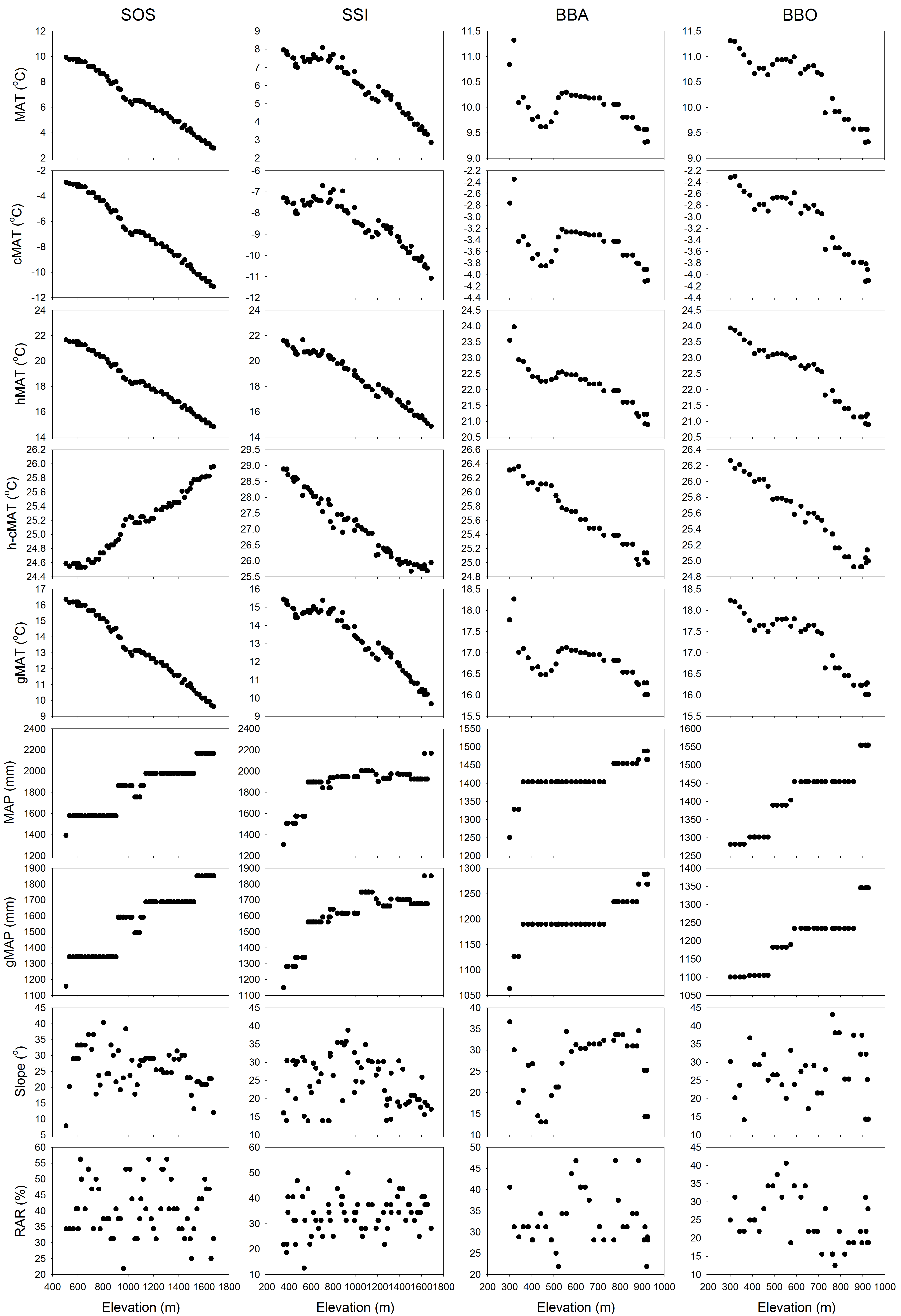

Supplement: S3 Fig — The abbreviations for environmental variables are as described in the S2 Table. (TIF) [file pone.0185763.s003.TIF]
