# Supplementary material for: Disentangling the local-scale drivers of taxonomic, phylogenetic and functional diversity in woody plant assemblages along elevational gradients in South Korea
Source: PLoS One. 2017 Oct 2;12(10):e0185763. doi: 10.1371/journal.pone.0185763 (PMC5624625; doi:10.1371/journal.pone.0185763)
Supplement: S2 Table — (DOCX) [file pone.0185763.s005.docx]

**S2 Table. The results of the principal components analysis with seven climatic variables.**

|  | **PC1** | **PC2** | **PC3** | **PC4** | **PC5** | **PC6** | **PC7** |
| --- | --- | --- | --- | --- | --- | --- | --- |
| Explained variance (%) | 81.60 | 15.27 | 3.01 | 0.06 | 0.05 | < 0.01 | < 0.01 |
| Loadings |  |  |  |  |  |  |  |
| MAT | 0.41 | 0.01 | 0.30 | –0.25 | –0.14 | 0.81 | 0.00 |
| cMAT | 0.41 | –0.20 | 0.26 | 0.42 | 0.19 | –0.14 | 0.70 |
| hMAT | 0.40 | 0.21 | 0.28 | 0.48 | 0.23 | –0.12 | –0.65 |
| gMAT | 0.41 | 0.12 | 0.30 | –0.61 | –0.21 | –0.55 | 0.00 |
| h-cMAT | –0.08 | 0.95 | –0.01 | 0.05 | 0.03 | 0.06 | 0.29 |
| MAP | –0.40 | –0.01 | 0.61 | 0.28 | –0.62 | –0.04 | 0.00 |
| gMAP | –0.40 | –0.04 | 0.55 | –0.27 | 0.67 | 0.04 | 0.00 |

* Abbreviations: MAT – mean annual precipitation, cMAT – mean temperature in the coldest month (January), hMAT – mean temperature in the hottest month (August), gMAT – mean temperature of growing season (May–August), h–cMAT – difference in temperature between the hottest and coldest months (August and January), MAP – mean annual precipitation, gMAP – mean precipitation of growing season
