# Supplementary material for: Disentangling the local-scale drivers of taxonomic, phylogenetic and functional diversity in woody plant assemblages along elevational gradients in South Korea
Source: PLoS One. 2017 Oct 2;12(10):e0185763. doi: 10.1371/journal.pone.0185763 (PMC5624625; doi:10.1371/journal.pone.0185763)
Supplement: S3 Table — The explained variance was divided into three parts: (1) pure effect of climatic distance; (2) shared effect of climatic and habitat distances; and (3) pure effect of habitat distance. The abbreviations for transect and diversity index are as described in Tables 1 and 4. (DOCX) [file pone.0185763.s006.docx]

**S3 Table. Variation partitioning of the beta diversity components explained by climatic and habitat distances** **along the four elevational transects.** The explained variance was divided into three parts: (1) pure effect of climatic distance; (2) shared effect of climatic and habitat distances; and (3) pure effect of habitat distance. The abbreviations for transect and diversity index are as described in Tables 1 and 4.

|  |  | **Explanatory distance matrices** | | |
| --- | --- | --- | --- | --- |
| **Transect** | **Diversity index** | **Climate** | **Shared** | **Habitat** |
| SOS | TBD | 0.343 | 0.034 | 0.014 |
|  | PBD | 0.009 | 0.004 | 0.025 |
|  | FBD | 0.011 | 0.006 | 0.041 |
| SSI | TBD | 0.145 | 0.007 | 0.004 |
|  | PBD | 0.002 | < 0.001 | 0.002 |
|  | FBD | 0.02 | 0.003 | 0.01 |
| BBA | TBD | 0.087 | 0.014 | 0.008 |
|  | PBD | 0.023 | 0.005 | 0.01 |
|  | FBD | 0.001 | < 0.001 | 0.002 |
| BBO | TBD | 0.181 | 0.012 | 0.02 |
|  | PBD | 0.132 | 0.008 | 0.022 |
|  | FBD | 0.144 | 0.008 | 0.019 |
